# Silence without stress

**DOI:** 10.7554/eLife.22073

**Published:** 2016-11-11

**Authors:** David J Young, Nicholas R Guydosh

**Affiliations:** Laboratory of Biochemistry and Genetics, National Institute of Diabetes and Digestive and Kidney Diseases, National Institutes of Health, Bethesda, United States; Laboratory of Biochemistry and Genetics, National Institute of Diabetes and Digestive and Kidney Diseases, National Institutes of Health, Bethesda, United Statesnicholas.guydosh@nih.gov

**Keywords:** translation, unfolded protein response, ubiquitination, *S. cerevisiae*

## Abstract

Two mechanisms ensure that the mRNA encoding Hac1 protein, a transcription factor involved in the unfolded protein response, is only translated when it is needed.

**Related research article** Di Santo R, Aboulhouda S, Weinberg DE. 2016. The fail-safe mechanism of post-transcriptional silencing of unspliced *HAC1* mRNA. *eLife* **5**:e20069. doi: 10.7554/eLife.20069

Cells, much like humans, must deal with a variety of stresses during their daily lives and react appropriately. In the absence of stress, it is also important that cells do not waste valuable resources on unnecessary responses.

Many proteins are synthesized by ribosomes that are bound to the membrane that surrounds the endoplasmic reticulum. The new proteins are then transported into the endoplasmic reticulum and folded into their correct final forms by molecular chaperones. However, many factors can cause unfolded or misfolded proteins to accumulate within the endoplasmic reticulum, which stresses it. In budding yeast, cells respond to such stress by expressing a transcription factor called Hac1p. This transcription factor is then transported to the nucleus, where it directs the expression of additional chaperones and other factors that can alleviate the stress (Cox and Walter, 1996; Mori et al., 1996). Many parts of this pathway are conserved across eukaryotes.

To guarantee a speedy response to endoplasmic reticulum stress, the messenger RNA (mRNA) for *HAC1* is stored in a ‘silenced’ form in the cytoplasm where it can be quickly activated (Chapman and Walter, 1997; Kawahara et al., 1997). Now, in eLife, Rachael Di Santo, Soufiane Aboulhouda and David Weinberg of the University of California, San Francisco, report the results of elegant experiments that reveal more details about the nature of this silencing mechanism (Di Santo et al., 2016). In addition, they reveal an intricate fail-safe mechanism that ensures the destruction of any Hac1 protein that escapes silencing.

Most mRNA molecules have regions called introns that are removed in a process called splicing before the mRNA molecules are translated into proteins. *HAC1* mRNA contains an atypical intron region that, in the absence of endoplasmic reticulum stress, undergoes base-pairing interactions with its own 5’ untranslated region (5’UTR; Sidrauski et al., 1996; Sidrauski and Walter, 1997). During endoplasmic reticulum stress, a protein senses the accumulation of unfolded proteins in the endoplasmic reticulum and removes the *HAC1* intron in response. This process leads to the expression of the full-length Hac1 protein (Figure 1).

**Figure 1.**
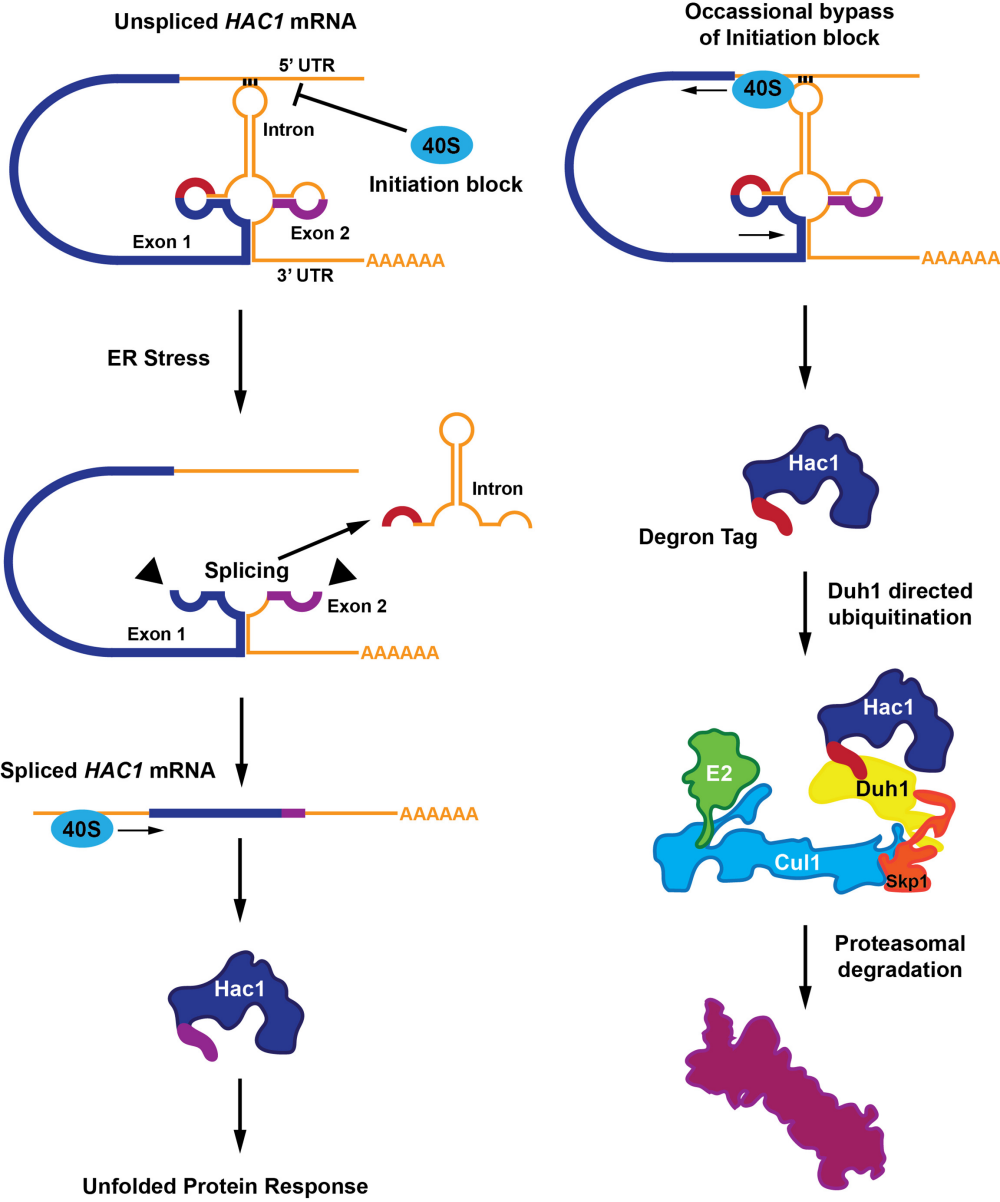
A functional Hac1 protein is made only if a block that prevents the initiation of translation is removed. (Left) The translation of two mRNA exons (shown here in blue and purple) results in the production of the Hac1 protein. However, unspliced *HAC1* mRNA contains an intron (orange) that, in the absence of endoplasmic reticulum stress, undergoes base-pairing interactions with its own 5’UTR. These interactions prevent most 40S ribosomes (blue oval) from loading onto the mRNA and initiating translation. When the endoplasmic reticulum becomes stressed (middle panel), Ire1 proteins (black triangles) splice out the intron. The spliced *HAC1* mRNA can then be translated to form the full-length Hac1 protein, which triggers the unfolded protein response in order to relieve endoplasmic reticulum stress. (Right) In the absence of stress, some 40S ribosomes (blue ovals) manage to bypass the base-pairing interactions. In this case, the second exon is not translated, and a new sequence is found at the C-terminal end of the protein (red). This "degron tag" is recognized by a protein adaptor called Duh1, which targets Hac1 for ubiquitination – a process that involves the formation of a complex that also includes E2, Cul1 and Skp1 proteins. This ubiquitination marks the protein for rapid degradation by the proteasome.

Two main models have emerged that attempt to explain how the *HAC1* mRNA is silenced by the base-pairing interactions between the intron and the 5’UTR. In the first model, the ribosomes are blocked by the interactions and cannot start translating the mRNA. In the second model the interactions cause the ribosomes to "stall" shortly after translation begins (Rüegsegger et al., 2001).

The stalled ribosome model was suggested by results from experiments that used a sucrose gradient to separate out the mRNA molecules that were being translated (and hence were attached to ribosomes) from those that were not. The results of these experiments showed that the majority of *HAC1* mRNAs were present in the polysome region of the sucrose gradient (where the majority of translation takes place). However, these results were called into question by ribosome-footprint profiling experiments by Weinberg and others that showed that ribosomes were nearly absent from *HAC1* transcripts in exponentially growing yeast (Weinberg et al., 2016). Through careful experimentation, Di Santo et al. reveal that the association of *HAC1* mRNA with polysomes in sucrose gradients was an artifact caused by non-specific interactions. Thus it appears that *HAC1* is silenced because ribosomes cannot start the translation process.

Di Santo et al. then uncovered an additional silencing mechanism that targets the Hac1 protein for destruction. In sucrose gradient experiments, mutating either the 5’UTR or the intron sequences in order to disrupt the base-pairing interaction resulted in the distribution of the mutant mRNAs being shifted to the polysome fractions. Although this suggested that translation could be initiated on these mutant transcripts, no protein was detectable. Further investigation revealed that the Hac1 protein synthesized from this mRNA variant was unstable because the intron encoded a 10-amino-acid tail that acted as a destruction signal (or “degron” tag).

Degron-mediated loss of the Hac1 protein occurs rapidly, making it difficult to record experimentally (Cox and Walter, 1996; Chapman and Walter, 1997; Kawahara et al., 1997). Di Santo et al. overcame this challenge with a number of clever biochemical tricks and genomic screens. These identified a previously unknown protein adapter, Duh1p, which recognizes the degron tag on Hac1 and targets the protein for ubiquitination, marking it for subsequent degradation by the proteasome.

The combination of inhibited translation initiation and accelerated protein degradation, both of which are dependent on the *HAC1* intron, provides exquisite control of Hac1 protein levels in the absence of endoplasmic reticulum stress. This finding raises the question of whether Duh1p may regulate other proteins and whether similar adaptors exist to regulate other degron tags in the cell. While the Hac1 protein was already known for the unusual way in which it is regulated, these discoveries add yet another plot twist to this fascinating story.
